# Evolving *Methanococcoides burtonii* archaeal Rubisco for improved photosynthesis and plant growth

**DOI:** 10.1038/srep22284

**Published:** 2016-03-01

**Authors:** Robert H. Wilson, Hernan Alonso, Spencer M. Whitney

**Affiliations:** 1 Research School of Biology, The Australian National University, Acton, Australian Capital Territory 2601, Australia

## Abstract

In photosynthesis Ribulose-1,5-bisphosphate carboxylase/oxygenase (Rubisco) catalyses the often rate limiting CO_2_-fixation step in the Calvin cycle. This makes Rubisco both the gatekeeper for carbon entry into the biosphere and a target for functional improvement to enhance photosynthesis and plant growth. Encumbering the catalytic performance of Rubisco is its highly conserved, complex catalytic chemistry. Accordingly, traditional efforts to enhance Rubisco catalysis using protracted “trial and error” protein engineering approaches have met with limited success. Here we demonstrate the versatility of high throughput directed (laboratory) protein evolution for improving the carboxylation properties of a non-photosynthetic Rubisco from the archaea *Methanococcoides burtonii*. Using chloroplast transformation in the model plant *Nicotiana tabacum* (tobacco) we confirm the improved forms of *M. burtonii* Rubisco increased photosynthesis and growth relative to tobacco controls producing wild-type *M. burtonii* Rubisco. Our findings indicate continued directed evolution of archaeal Rubisco offers new potential for enhancing leaf photosynthesis and plant growth.

Improving the performance of the CO_2_-fixing enzyme Rubisco has the potential to significantly enhance photosynthetic efficiency and yield^1^. Strategies to achieve this goal involve either modifying the biochemistry and ultrastructure of leaf chloroplasts to concentrate CO_2_ around Rubisco, or directly improving Rubisco catalysis itself by genetic crossing or transgenic modification^2^. While both approaches face significant technical challenges, suggestions that Rubisco in plants is already operating at or near physiological optimum poses uncertainty as to the level of improvement possible^3^,^4^. Somewhat overlooked in these small data set analyses is that plant Rubisco is not the pinnacle of evolution - as the superior Rubisco from some red algae have the potential to benefit C_3_-plant productivity by as much as 30%^2^. Unfortunately, replacing plant Rubisco with red algal Rubisco appears untenable due to chaperone incompatibilities that preclude assembly of algal Rubisco large (L-) and small (S-) subunits into functional L_8_S_8_ hexadecamer complexes in leaf chloroplasts^5^. In recent years there have been significant advances in understanding the complex and specialised ancillary chaperones for the biogenesis of cyanobacteria and plant L_8_S_8_ Rubisco^6^,^7^,^8^,^9^, however homologs for many of these chaperones in red algae are not readily identifiable.

Despite five decades of research, a dramatic amplification in computational power and more than 25 X-ray structures for different Rubisco isoforms^10^ we remain unable to improve Rubisco catalysis by rational design^11^,^12^,^13^. This limitation has led to the development of directed (*in vitro* or laboratory) protein evolution approaches tailored to select for Rubisco mutants with improved function^12^. In general, directed protein evolution involves the identification of proteins with desired properties from a mutant library comprising sufficient genetic diversity^14^. Advances in directed protein evolution technologies have spurred its success in identifying mutations that improve, or alter, the catalysis and/or solubility of a diverse array of enzymes^14^,^15^,^16^. A key benefit of directed evolution is it can reveal novel fitness solutions that would likely otherwise go unexplored during natural evolution^14^,^17^.

Directed evolution of Rubisco has primarily used low throughput photosynthetic selection systems (e.g. *Rhodobacter capsulatus*) or high throughput Rubisco dependent *E. coli* (RDE) selection systems that vary dramatically in efficiency^12^,^18^,^19^. Common to RDE selection systems is the ectopic expression of phosphoribulokinase (PRK) whose product, the 5-carbon substrate of Rubisco ribulose-1,5-bisphospahte (RuBP), is fortuitously toxic to bacteria. A refined MM1-*prk* RDE selection has been genetically tailored to use a ‘PRK-Rubisco shunt’ to bridge a *gap*A^−^ introduced break in glycolysis (Fig. 1a 20,21). The low frequency of false positives obtained using the MM1-*prk* RDE system contrasts with the striking inefficiency of other RDE systems^22^,^23^. As a result, the MM1-*prk* RDE system has identified mutations that negatively influence the CO_2_/O_2_ specificity (S_C/O_) of bacterial L_2_ Rubisco from *Rhodospirillum rubrum* as well as mutations that significantly enhance the assembly (solubility) of cyanobacteria L_8_S_8_ Rubisco and, in one instance, marginally improved all catalytic parameters^12^,^24^. More recently, the MM1-*prk* RDE identified a *Synechocystis* PCC6803 L_8_S_8_ Rubisco mutant with 3-fold improvements in carboxylation efficiency that improved photosynthesis rates by >50% when re-integrated into the high CO_2_ carboxysome compartment within the cyanobacterium^11^. In contrast, it is unlikely that these improvements would be of benefit in plant leaves as the high CO_2_ levels needed to account for the low CO_2_ affinity and poor S_C/O_ of cyanobacteria Rubisco are not met by chloroplasts^25^.

The non-photosynthetic role of the ancient L_2_/L_10_ Rubisco isoforms in archaea implies they have likely undergone alternative selection pressures to photosynthetic Rubisco during evolution. For example, Rubisco from archaea have a high affinity for RuBP and high thermostability but low carboxylation rates (*k*_*cat*_^*C*^) and S_C/O_^26^,^27^,^28^. This catalytic distinctiveness arises from the alternative biological role of archaeal Rubisco in the pentose bisphosphate pathway where it functions to metabolize the RuBP produced during nucleoside metabolism^29^. Despite its non-photosynthetic function, archaeal Rubisco can still support plant photosynthesis and growth. For example, the *Methanococcoides burtonii* L_10_ Rubisco (MbR) is highly expressed in leaf chloroplasts and shown to support the growth to fertile maturity of tobacco under the high CO_2_ levels needed to accommodate the low *k*_*cat*_^*C*^ and S_C/O_ of MbR^26^.

The significantly poorer carboxylase properties and alternative function of Rubisco in archaea suggest this form of the enzyme has adapted to alternative evolutionary pressures compared with Rubisco in photosynthetic organisms. This questions whether the carboxylation properties of the archaeal L_10_ Rubisco might be more amenable for improvement towards those required for enhanced photosynthetic potential. To address this question we used the MM1-*prk* RDE system (Fig. 1a) to select evolved MbR mutants with improvements in catalytic properties that are required to enhance C_3_-plant photosynthesis^5^,^30^. These properties include increasing *k*_*cat*_^*C*^, carboxylation efficiency (*k*_*cat*_^*C*^ divided by *K*_*C*_^*21%O2*^; the *K*_*m*_ for CO_2_ under ambient O_2_) and S_C/O_. Using chloroplast genome (plastome) transformation we introduce *mbR* genes into tobacco to demonstrate successful translation of improved MbR properties selected in *E. coli* into leaf chloroplasts. The enhanced photosynthesis and growth of the transformed plants producing improved MbR mutants relative to control lines producing non-mutated MbR provides novel proof of concept on the utility of improving Rubisco catalysis by directed evolution in *E. coli* to improve the CO_2_-assimilation rate in leaves.

## Results

### Directed evolution of *M. burtonii* Rubisco (MbR) in *E. coli*

The native *mbii*L gene coding *M. burtonii* Rubisco is efficiently translated in *E. coli* and assembles into abundantly expressed (>6% (w/w) of soluble cell protein) as functional L_2_ Rubisco (MbR)^26^. In the presence of substrate RuBP (or structurally comparable sugar phosphate ligand) the L_2_ units assemble into a stable L_10_ MbR complex. Random mutations were introduced into *mbii*L using error prone PCR (averaging 2 mutations per kb) and the mutant genes ligated into a *lac* inducible vector pTrcHisB^20^.

**Figure 1:**
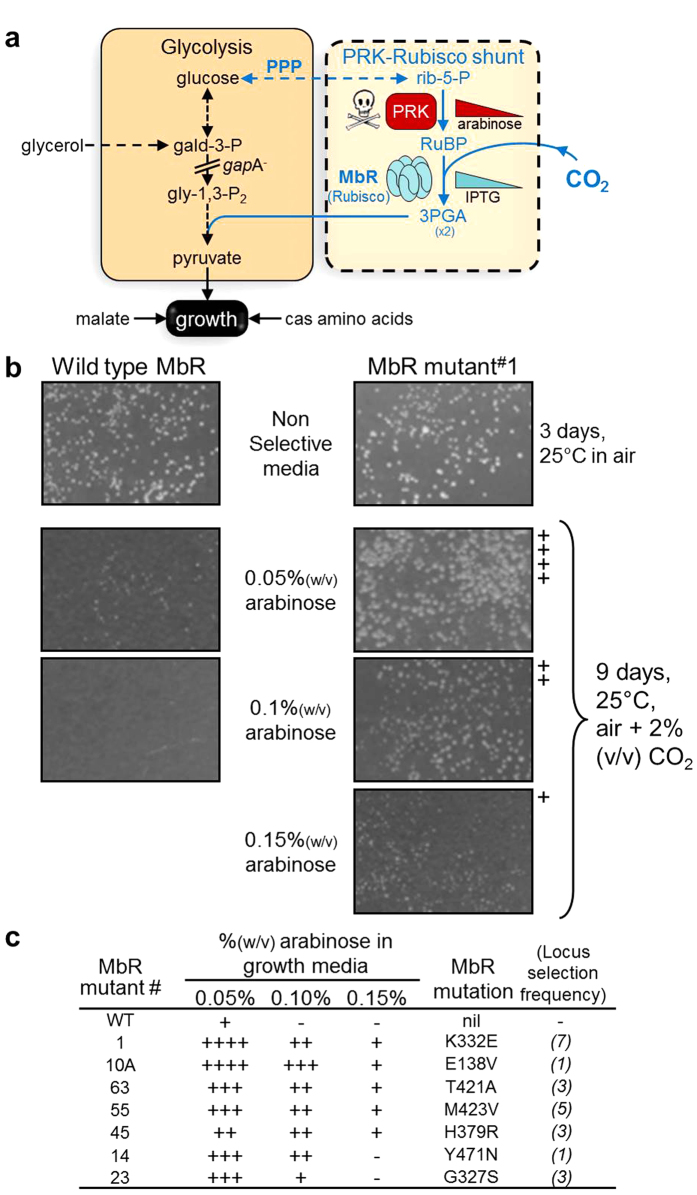
Selecting for improvements in MbR catalysis using Rubisco Dependent *E. coli* (RDE). (**a**) Simplified schematic of the RDE selection system that uses a glycolysis/gluconeogenesis interrupted glyceraldehyde-3-phosphate dehydrogenase deletion (*gap*A^−^) strain of *E. coli* (MM1). Ectopic expression of phosphoribulokinase (PRK) and Rubisco in MM1 acts as a bypass shunt for glycolysis to enable carbon flow from hexose carbon to the TCA cycle for energy and growth. PRK catalyses the conversion of ribulose-5-phosphate (rib-5-P) produced by the pentose phosphate pathway (PPP) to RuBP, which is toxic to cell growth. RuBP toxicity can be alleviated by Rubisco catalysed carboxylation followed by utilisation of the 3PGA product in glycolysis. Expression of PRK and Rubisco is carefully regulated by varying the inducing agents arabinose and IPTG respectively^20^. Early cell division is facilitated by addition of trace levels of glycerol (0.4% v/v; upstream C-source), 30 mM malate and 0.5% w/v cas amino-acids (downstream C- and N- sources). (**b**) Comparative growth of MM1-*prk* cells expressing “improved” mutant and wild type MbR under increasing levels of arabinose induced PRK expression. (−) no growth; (+ to ++++) relative increase in colony size. See Supplementary Table 1 for comprehensive RDE growth screen and sequence of all primary MbR mutants selected. (**c**) A summary of colony growth scores for the seven best performing MbR mutants under increasing arabinose induced PRK expression.

Three *mbii*L libraries (each comprising ~180 k variants) were transformed into MM1-*prk* cells (Fig. 1a) and grown at 23 °C as described^20^. The initial selection was performed under high-Rubisco inducing (0.5 mM IPTG) and low-PRK inducing conditions (0.05% (w/v) arabinose) in air supplemented with 2.5% (v/v) CO_2_. After 9–16 days 80 colonies showing improved growth relative to MM1-*prk* producing wild-type MbR were identified (Supplementary Table 1). The Rubisco-containing plasmid from each colony was sequenced revealing substitutions in 78 of the 474 amino acids (Supplementary Table 1). Each *mbii*L mutant was cloned back into pTrcHisB and re-transformed into MM1-*prk* RDE cells and separately grown under higher Rubisco activity selection (*i.e.* on media containing 0.1% (w/v) arabinose to elevate PRK expression; Fig. 1b). Colony growth was scored relative to MM1-*prk* cells expressing wild-type MbR that could not grow on media containing 0.1% (w/v) arabinose (Fig. 1b). Seven *mbii*L mutant genes were found to convey a distinct selective advantage to MM1-*prk E. coli* growth (Fig. 1c).

**Figure 2:**
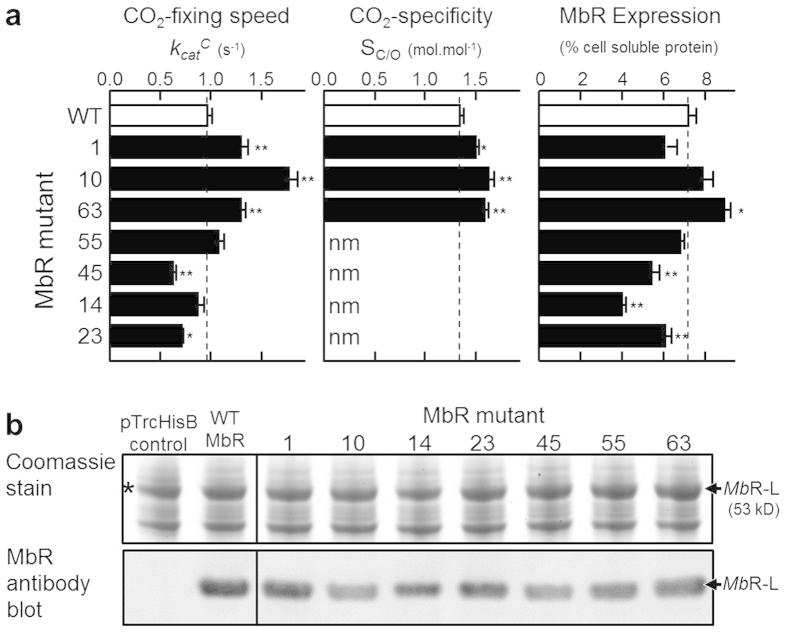
Analysis of MbR mutants with improved catalysis. (**a**) Catalytic properties (maximum RuBP-carboxylation rate, *k*_*cat*_^*C*^; specificity for CO_2_ over O_2_, S_C/O_) of wild type and mutant MbR at 25^o^C, pH 7.2 and their relative expression level in crude *E. coli* soluble lysate. Values shown are the average (±S.E) of assays made on three cell preparations or, for S_C/O_, four technical replicates from duplicate purified enzyme preparations. nm, not measured. Significance variation relative to wild type MbR (*p < 0.01, **p < 0.001) determined by T-test. (**b**) SDS PAGE analysis and immuno-blot detection of MbR content in the *E. coli* soluble protein (7 μg/lane) used to measure *k*_*cat*_^*C*^ and MbR content. pTrcHisB, vector-only control, *an abundant ~50kDa protein made in *E. coli* that is of similar size to the Rubisco L-subunit^21^.

The seven mutant and wild-type pTrc-*mbii*L genes were expressed in XL1-Blue *E. coli* without co-expressing PRK. This resulted in only L_2_ MbR oligomers being formed as the cells made no RuBP that is required for the formation of L_10_ MbR complexes^26^. The cellular content and catalytic properties of each L_2_ MbR enzyme was measured (Fig. 2a). Maximal rates of CO_2_-fixation (*k*_*cat*_^*C*^) at 25 °C were determined under ambient O_2_ levels (~252 μM O_2_) and at pH 7.2 due to the low pH favoured by MbR catalysis^26^. Under these conditions MbR mutant isolates #1 (MbR-K332E), #10A (MbR-E138V) and #63 (MbR-T421A) showed significant 40% to 90% improvements in *k*_*cat*_^*C*^ and corresponding 10% to 25% increases in S_C/O_ (Fig. 2a). Quantification of MbR expression in *E. coli* by ^14^C-CABP binding and confirmation by SDS PAGE (Fig. 2b) showed that most mutations had little effect on the level of MbR expression. The MbR-T421A mutant (#63) showed a modest, but significant, increase in expression while the mutations in MbR mutants #14, #23 and #45 significantly impeded MbR production (Fig. 2b).

### Expression of improved MbR in tobacco chloroplasts

**Figure 3:**
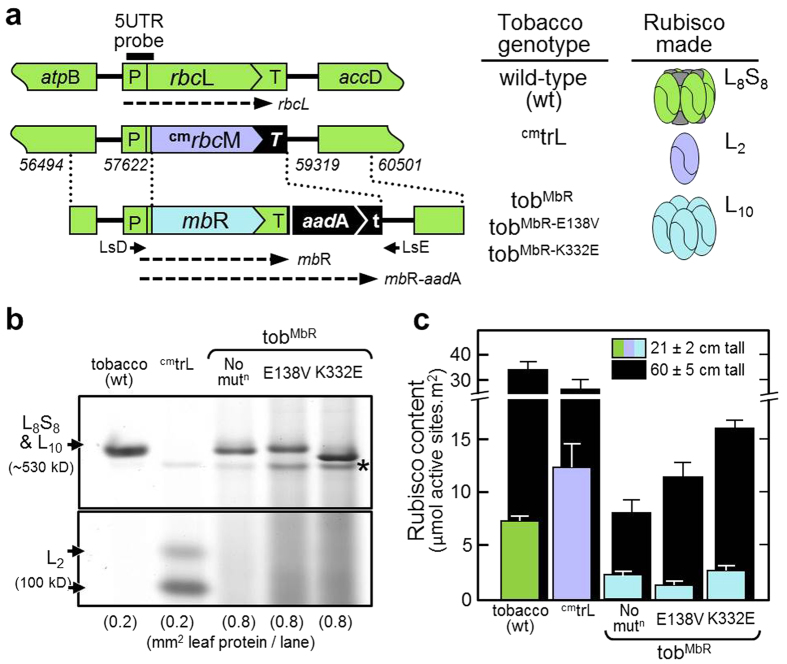
Transformation and expression of the mutated MbR enzymes in tobacco leaves. The varying ^cm^*mb*R genes coding wild type and mutant MbR were integrated into the *rbc*L region of the tobacco plastome by chloroplast transformation. (**a**) Comparison of the plastome sequence and types of Rubisco made in the varying tobacco genotypes examined. The ^cm^*mb*R and selectable marker *aad*A gene in the pLEVMbR, pLEVMbR-E138V and pLEVMbR-K332E transforming plasmids were transformed into the plastome of the ^cm^trL tobacco genotype to replace the ^cm^*rbc*M (that codes *R. rubrum* L_2_ Rubisco^31^) by homologous recombination of the flanking plastome sequence (located between the dashed lines, numbering relevant to Genbank sequence Z00044). P, 292-bp *rbc*L promoter/5′UTR; T, 288-bp*rbc*L 3′UTR; *T*, 112-bp of *psb*A 3′UTR; t, 147-bp *rps*16 3′UTR. Alignment position for primers LsD and LsE^32^ and the 221-bp 5UTR probe^8^ are shown. (**b**) native PAGE analysis of the L_8_S_8_, L_2_ and L_10_ Rubisco isoforms produced, respectively, in leaves from tobacco, ^cm^trL and the three tob^MbR^ genotypes. *non-Rubisco protein. (**c**) ^14^C-CABP quantification of Rubisco active site content in comparable young upper canopy leaves of each genotype during early (~20 cm in height, colored bars) and late (~60 cm in height, black bars) exponential growth.

The tobacco genotype ^cm^trL has been genetically tailored for Rubisco engineering using chloroplast transformation^31^. In the plastome of ^cm^trL chloroplasts the wild type *rbc*L gene has been replaced with a synthetic, codon modified version of the *R. rubrum* bacterial *rbc*M gene (^cm^*rbc*M) that codes for Form II L_2_ Rubisco in place of tobacco L_8_S_8_ Rubisco (Fig. 3a). The increased O_2_ sensitivity of *R. rubrum* L_2_ Rubisco reduces both S_C/O_ and carboxylation efficiency under ambient O_2_ (*k*_*cat*_^*C*^/*K*_*C*_^*21%O2*^) by ~7-fold relative to tobacco L_8_S_8_ Rubisco (Table 1). These poorer catalytic properties result in the ^cm^trL genotype requiring high CO_2_ for growth in soil^31^. As shown by Alonso *et al.*, (2009) the catalytic properties of MbR (both in L_2_ and L_10_ complexes) are even more impeded than *R. rubrum* L_2_ Rubisco, especially with increasing alkaline pH. Despite this impairment, transplastomic replacement of the ^cm^*rbc*M gene in ^cm^trL with the wild-type *mbii*L gene generated the L_10_ MbR producing tobacco genotype tob^*mbii*L^ that could survive under elevated CO_2_ (2.5% v/v) in soil^26^. The tob^*mbii*L^ lines took more than 300 days to reach fertile maturity compared with ~30 days for wild type tobacco and ~32 days for ^cm^trL under the same growth conditions.

To test whether the evolved MbR enzymes translated to improved tobacco photosynthesis and growth, synthetic *mb*R genes were made that incorporated the codon use of the tobacco *rbc*L gene (Fig. 3a). In addition, the native MbR N-terminal MSLIYEDLV sequence was replaced with the MSPQTETKASVGF sequence of the tobacco L-subunit that undergoes a range of post-translational modifications that tentatively provide protection from proteolysis^13^. Three *mb*R genes coding wild-type MbR, MbR-K332E and MbR-E138V were cloned into the pLEV4 plastome transforming plasmid and transformed into ^cm^trL leaves^31^. Transplastomic tobacco lines producing L_10_ MbR were identified by native PAGE (Fig. 3b). At least two independent lines for each of the tob^MbR^, tob^MbRE138V^ and tob^MbRK332E^ genotypes were continuously propagated on spectinomycin-containing media until homoplasmic (*i.e.* no longer producing L_2_ *R. rubrum* Rubisco) before growing the T_0_ plants to maturity in soil in air supplemented with 2.5% [v/v] CO_2_.

Only L_10_ MbR was detected in leaves (Fig. 3b) due to the continuous production of RuBP under illumination and the relative stability of the decameric complex^26^. While the T_0_ tob^MbR-E138V^ and tob^MbR-K332E^ plants grew substantially quicker than tob^MbR^, little difference was detected in the L_10_ MbR content in comparable upper canopy leaves of the juvenile (~21 cm tall) T_0_ plants (Fig. 3c). When at ~60 cm in height, 3–6-fold higher levels of L_10_ MbR were measured in the newly emerging upper canopy leaves with significantly higher amounts detected in the faster growing, healthier looking, tob^MbRE138V^ and tob^MbRK332E^ T_0_ plants (Fig. 3c). At both development stages, the leaf MbR levels were generally 3–4-fold lower than the L_2_ and L_8_S_8_ Rubisco content in the ^cm^trL and wild-type tobacco controls growing alongside.

Limitations in the steady state *mb*R mRNA levels in each genotype contributed to the deficiency in MbR (Supplementary Fig. 1). As indicated in Fig. 3a, both a monocistronic *mb*R and a (50–70% less abundant) discistronic *mb*R-*aad*A transcript were made in each T_0_ tob^MbR^ genotype. In the young upper leaves of T_0_ plants at ~21 cm in height the total *mb*R mRNA abundance was 30–70% lower in abundance than the *rbc*L mRNA levels in wild-type (Supplementary Fig. 1). As seen previously in Rubisco-modified tobacco genotypes with reduced photosynthetic potential^8^,^26^,^30^,^32^, these reduced mRNA levels correlate with the impaired viability of the thinner, smaller sized, pale green leaves of each transplastomic genotype (see below).

### The evolved MbR have improved carboxylase activity

The catalytic properties of the wild type and mutant L_10_ MbR isoforms produced in the T_0_ progenies were measured at pH 8 (the approximate pH of the chloroplast stroma, Table 1). While the S_C/O_ values matched those measured for the L_2_ enzymes produced in *E. coli* (Fig. 2b), the *k*_*cat*_^*C*^ rates were lower than those measured at pH7.2 due to the increased activity of MbR at low pH^26^. Nevertheless, even at pH 8 both *k*_*cat*_^*C*^ and the carboxylation efficiencies (*k*_*cat*_^*C*^/*K*_*C*_^*21%O2*^) of the MbR-E138V and MbR-K332E enzymes were between 2 and 3.4-fold higher than MbR, with an accompanying ~15% increase in S_C/O_ for the MbR-E138V enzyme (Table 1). Importantly, these improvements in CO_2_ affinity, specificity and fixation speed came without expense to the natural high affinity of MbR for RuBP (*i.e*. a low *K*_*m*_^*RuBP*^, Table 1).

### Enhancing MbR catalysis improves tobacco photosynthesis and growth

**Figure 4:**
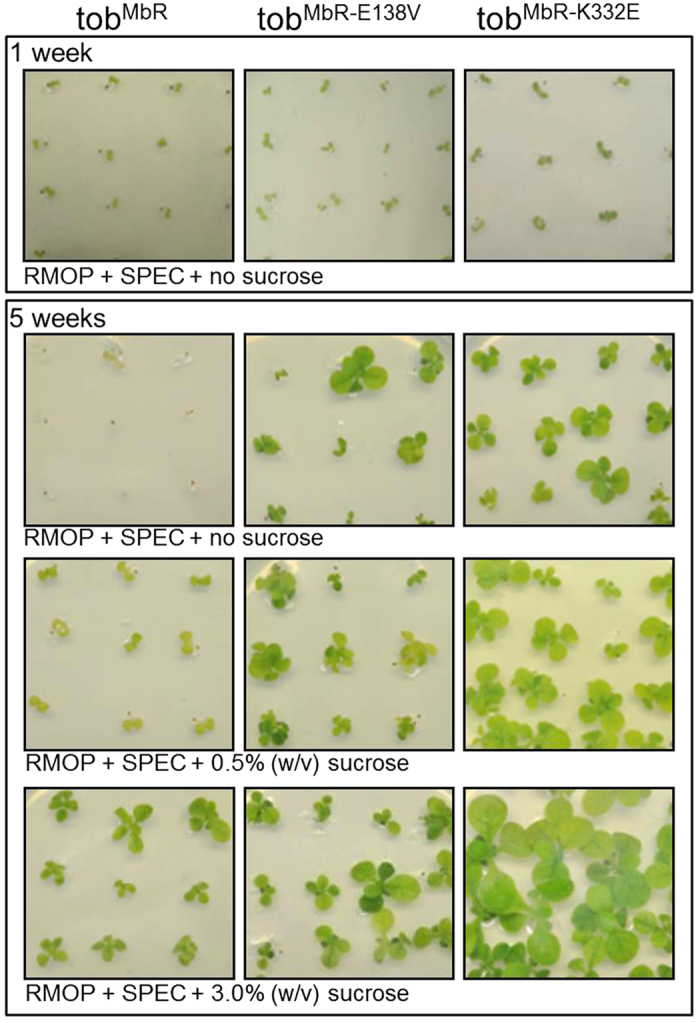
The T_1_ tob^MbR-E138V^ and tob^MbR-K332E^ progeny showed improved growth in tissue culture. Comparison of the growth of each tob^MbR^ genotype after 1 and 5 weeks growth on RMOP media containing 0.2 mg.mL^−1^ spectinomycin and varying levels of sucrose. The plants were grown in air +2% (v/v) CO_2_ and 25–100 μmol photons.m^−2^.s^−1^ illumination.

The improved growth and healthier phenotype of the tob^MbRK332E^ and tob^MbRE138V^ genotypes relative to the tob^MbR^ lines was evident in the T_1_ progeny. In tissue culture germination trials all the T_1_ progeny emerged as green cotyledons on spectinomycin media after 1 week confirming all were transplastomic (Fig. 4). After 5 weeks it was evident that addition of sucrose to the tissue culture media was required for the germinated tob^MbR^ and tob^MbRE138V^ lines to survive under elevated (2.5% v/v) CO_2_ (Fig. 4). In contrast, the tob^MbRK332E^ plants survived under high CO_2_ without sucrose supplementation and grew quicker under all tissue culture conditions tested.

**Figure 5:**
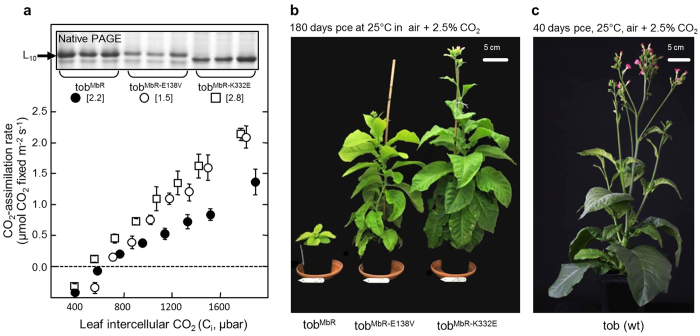
The mutated MbR improve tobacco leaf photosynthesis and plant growth relative to tobacco producing wild-type MbR. (**a**) Comparison of photosynthesis rates in plants growing in soil quantified by leaf gas exchange measures of CO_2_-assimilation rates at 25 °C at varying intercellular CO_2_ pressures (C_i_). Shown are the average of 3 measurements (±S.D) made at 1000 μmol quanta m^−2^ s^−1^ illumination on comparable upper canopy leaves of T_1_ plants 21 ± 2 cm in height. The L_10_ Rubisco content in the analysed leaves were quantified by ^14^C-CABP binding (the average μmol catalytic sites.m^2^ shown in square brackets) and confirmed by coomassie staining following native PAGE. (**b**) A growth comparison of the transplastomic tobacco genotypes after 180 days at high CO_2_ highlighting the faster growth of the tob^MbR-E138V^ and tob^MbR-K332E^ plants relative to the tob^MbR^ controls, albeit slower than (**c**) the 40 day old wild type tobacco. pce, post-cotyledon emergence.

As shown by Alonso *et al.*, (2009), air enriched with >2% (v/v) CO_2_ was needed for each MbR producing tobacco line generated to grow to fertile maturity in soil. Consistent with the improved catalysis of the transplanted MbR-E138V and MbR-K332E enzymes (Table 1), the tob^MbRE138V^ and tob^MbRK332E^ genotypes supported faster leaf photosynthetic CO_2_ assimilation rates relative to the tob^MbR^ lines (Fig. 5a). To compensate for the lower leaf levels of MbR and the poorer catalytic properties of the L_10_ MbR relative to tobacco L_8_S_8_ Rubisco (Fig. 3c and Table 1), measurements of photosynthetic CO_2_-assimilation rates in all the MbR transformed leaves were performed under 1% (v/v) O_2_. Even under these low O_2_ pressures, photosynthesis remained limited by MbR-activity over the full range of intercellular leaf CO_2_ pressures (C_i_) tested (Fig. 5a) with the highest assimilation rate of 2.4 μmol CO_2_ fixed.m^2^.s^−1^ measured in tob^MbRK332E^ leaves under the highest leaf gas exchange C_i_ of 2000 μbar CO_2_ (Fig. 4b). As this rate is more than 10-fold slower than the 26–30 μmol CO_2_ fixed.m^2^.s^−1^ rates measured in high CO_2_ grown wild type leaves^33^ the tob^MbRK332E^ grew ~5-fold slower than wild-type under high CO_2_ (Fig. 5b,c).

The relative differences in the leaf CO_2_-assimilation rates of each transplastomic genotype (Fig. 5a) correlated with their growth rate (Fig. 5b). The tob^MbRK332E^ plants grew faster and reached fertile maturity before the tob^MbRE138V^ lines while the growth of the tob^MbR^ controls were substantially impaired (Fig. 5b). The faster growth by the tob^MbRK332E^ plants contrasts with the better carboxylation properties of MbR-E138 V relative to MbR-K332E (Table 1). This discrepancy can be attributed to the ~50% lower levels of MbR-E138 V produced in tob^MbRE138V^ leaves relative to MbR levels produced in comparable leaves from both the tob^MbR^ and tob^MbRK332E^ genotypes (Fig. 5a). Identifying if the E138 V mutation impedes the translation, biogenesis or/and stability of MbR remains to be tested.

### The structural location of the E138 V and K332E mutations in MbR

**Figure 6:**
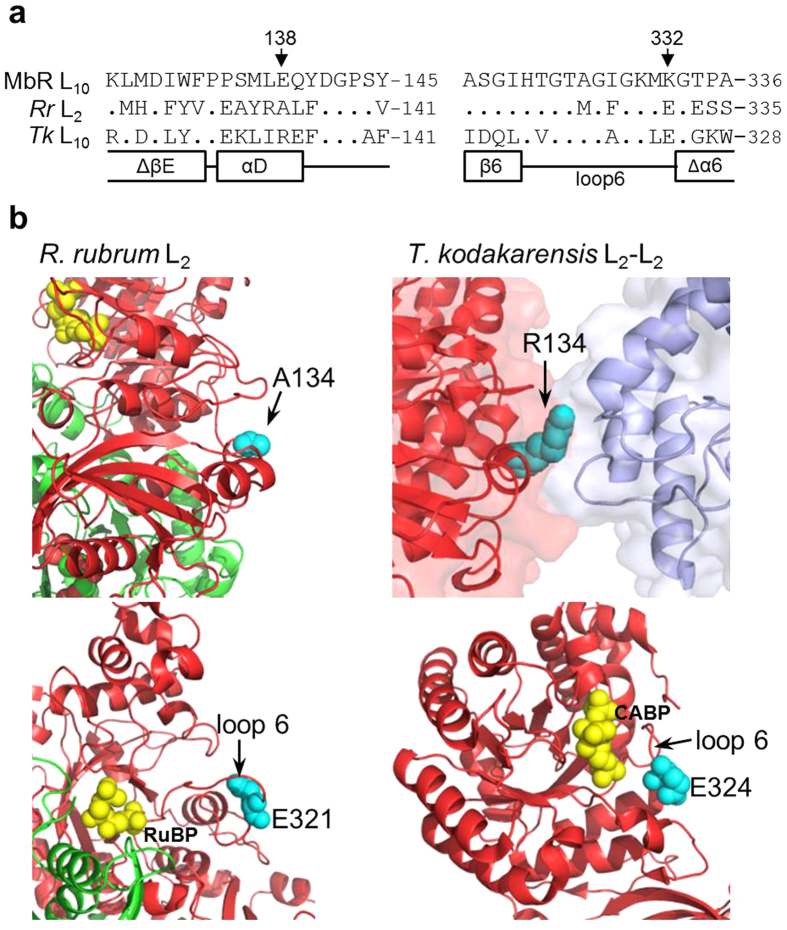
Structural analysis of the catalysis enhancing mutations in MbR. (**a**) Alignment of MbR, *R. rubrum* (*Rr*) and *T. kodakorensis* (*Tk*) Rubisco large subunit sequences adjoining the E138 and K332 mutation sites in MbR. Only amino acids differing from MbR are shown. Secondary structure information is relative to that in *Tk* L_10_ Rubisco^27^. (**b**) Location of the mutated residues in *R. rubrum* L_2_ Rubisco (A134 and E321: PDB 9RUB) and *Tk* L_10_ Rubisco (R134 and E324: PDB 3A12) are highlighted in cyan. *Rr* L-subunits are shaded red and green, active site bound RuBP or CABP in yellow. In the *Tk* structure R134 is located at the L_2_-L_2_ interface (differentially coloured red and blue). E321 and E324 are located within the flexible loop 6 region in both *Rr* and *Tk* Rubisco respectively. Diagrams constructed using PyMOL.

Phylogenetic analysis of MbR reveals it shares closer sequence homology with *R. rubrum* Form II Rubisco than other archaeal Rubisco (Supplemental Fig. 2)^26^. These alignments showed that E138 and K332 in MbR align with A134 and E331 in *R. rubrum* Rubisco and R134 and E324 in the *T. kodakorensis* archaeal L_10_ Rubisco (Fig. 6a). As shown in Fig. 6b, A134 in the *R. rubrum* L_2_ crystal structure is solvent exposed and located distil to the active site. In contrast R134 in the *T. kodakarensis* L_10_ structure is one of only ten amino acids that form a highly ionic network between adjoining dimers (*i.e.* at each L_2_-L_2_ interface)^27^.

In both *R. rubrum* and *T. kodakorensis* Rubisco, the corresponding E331 and E324 residues are located near the hinge of the conserved flexible loop 6 structure of the C-terminal α/β-barrel (Fig. 6b). A glutamate at this position in loop 6 is highly conserved among photosynthetic L_8_S_8_ Rubisco isoforms (e.g. E336 in plants like tobacco, E339 in red algae such as *Griffithsia monilis*) and is in close vicinity to the strictly conserved K334 catalytic residue (tobacco Rubisco numbering) whose side-chain interactions with RuBP and gaseous substrate are critical determinants of catalytic efficiency (*i.e. k*_*cat*_^*C*^ and S_C/O_)^10^. The increased catalytic turnover rate and carboxylation efficiency of MbR-K332E (Table 1) imply a glutamate at this position in loop 6 may benefit Rubisco catalysis in photosynthetic organisms but pose no benefit for the non-photosynthtic role of archaea Rubisco.

## Discussion

He we uniquely demonstrate the potential of directed evolution using RDE selection to successfully deliver more efficient forms of the non-photosynthetic *M. burtonii* archaeal Rubisco (MbR). The derived improvements in CO_2_-fixation speed, CO_2_-affinity and specificity for CO_2_ of the evolved MbR-E138 V and MbR-K332E mutant enzymes translated to supporting faster rates of CO_2_ assimilation and growth in tobacco relative to the control tob^MbR^ genotype producing wild-type MbR. This finding provides the first proof of concept that directed evolution of non-photosynthetic Rubisco in *E. coli* can deliver mutants with improvements in all the catalytic parameters needed to stimulate photosynthesis in leaf chloroplasts. This contrasts with prior success in evolving improved catalytic mutants of cyanobacterial Rubisco that either show only marginal (<5%) overall improvements in catalysis^24^ or a significant enhancement (>50%) in carboxylation efficiency that came at the expense of an unwanted parallel increase in inhibitory oxygenation efficiency^11^. An additional challenge with cyanobacterial L_8_S_8_ Rubisco is its limited biogenesis potential in tobacco chloroplasts (~10% of wild-type^34^) compared with MbR L_10_ which is produced at ~25–50% of wild-type tobacco Rubisco (Fig. 3c). The high solubility and overall success with evolving MbR catalysis inspires continued effort to evolve properties along evolutionary trajectories that further enhance its photosynthetic potential.

Exploration of Rubisco sequence space towards mutations that improve its efficiency in crop plants is an ongoing challenge^13^. Our continued inability after 50 years to rationally predict what sequence changes can improve Rubisco function steered our attention towards the potential of directed evolution to explore Rubisco sequence space for improved catalysis. A common requirement of successful directed evolution studies is identifying a suitable starting point for mutagenesis and appropriate selection system^14^,^17^,^35^. The ease by which the carboxylase activities of MbR could be enhanced by single amino acid changes (Table 1) likely stems from it having undergone specialisation to an alternative metabolic role during its non-photosynthetic evolution^29^. This implies that archaeal Rubisco may occupy an alternative position to photosynthetic Rubisco within the evolutionary landscape of sequence space diversity in relation to catalysis. Consistent with this, archaeal Rubisco catalysis is typically distinct relative to contemporary (photosynthetic) Rubisco^26^. Archaeal Rubisco can sustain functionality at extreme temperatures, under which thermotolerant archaea grow, and exhibit the heightened affinity for RuBP required to metabolise the finite levels made during nucleotide metabolism^29^. Offsetting these beneficial features, archaeal Rubisco show low *k*_*cat*_^*C*^ and S_C/O_^26^. Improving our fundamental understanding of the structural features that determine these unconventional kinetics requires a more comprehensive survey of archaeal Rubisco structure, catalytic, and sequence diversity.

The merits of directed protein evolution are illustrated by the many examples of successfully altering protein solubility, improving catalysis, even enabling promiscuous catalytic function^12^,^14^,^15^,^17^. These outcomes typically require multiple, incremental rounds of mutagenesis. For MbR, an attempt was made to select second generation mutants with improved activity using a combined library of epPCR mutated *mb*R genes coding MbR, MbR-E138V and MbR-K332E. No MbR mutants were detected that enabled MM1-prk RDE survival under high PRK induction (*i.e.* 0.2% w/v arabinose). Future goals are to examine the feasibility of capturing the epistasis of the spectrum of first generation *mbii*L mutant genes (Supplementary Table 1) using a shuffling approach to identify adaptive trajectories that further improve MbR catalysis. Success however depends on the positive epistatic potential for evolving the carboxylase activity of MbR and ability to avoid or circumvent beneficial mutations that might produce destabilizing effects on structure and function. Such uncertainties are common to directed protein evolution studies. Forecasting the extent to which mutations (via direct or long distance amino acid interactions) influence artificial evolutionary trajectories remains unpredictable^36^.

A significant hurdle is the relatively low selection fidelity and throughput of the MM1-*prk* selection system^20^. The frequency of success in directed evolution applications depends on the library selection throughput and sensitivity of the selection system to detect a desired trait^14^,^15^. The reliance and throughput of existing RDE strains suffer from high frequencies of false positives that typically arise through transposon associated PRK escape mutations^11^,^12^. While relatively immune to false positives, the MM1-*prk* RDE selection throughput is impeded by a low growth temperature requirement (25 °C), poor transformation efficiency, and reduced cell viability as a result of the *gap*A^−^ mutation^20^. Improving the selection fidelity of RDE systems is therefore critical to further evolving MbR, and other Rubisco isoforms, with improved photosynthetic properties. One solution might be to tether PRK with an antibiotic resistance protein in an RDE strain thus avoiding selection of “PRK-silenced” false positives as such mutations would also relinquish antibiotic resistance.

Adaptive evolution of archaeal Rubisco *in vitro* towards one that is more efficient than crop plant L_8_S_8_ enzymes is undeniably a significant, long term challenge. Unlike L_8_S_8_ Rubisco from plants and algae, the folding and assembly requirements of archaeal Rubisco, like MbR, are met in *E. coli* (Fig. 1c)^26^,^27^,^29^. This property strengthens the suitability of archaeal Rubisco for identifying catalysis enhancing mutants using RDE strains as it curtails selection of mutations that enhance solubility, an outcome that has dominated directed evolution studies with cyanobacteria L_8_S_8_ Rubisco^11^,^12^,^22^. The amenability of archaeal Rubisco to mutational testing in *E. coli* has already proven useful to demonstrate how incorporating spinach Rubisco sequence into *T. kodakarensis* L_10_ Rubisco can improve *k*_*cat*_^*C*^ 27. Improving archaeal Rubisco catalysis by rational design or directed evolution or a combination of both therefore poses viable future pathways to pursue, particularly given our finding that these benefits can directly translate to improving leaf photosynthesis.

As indicated in Fig. 6, the primary sequences of archaeal Rubisco are highly diverse and their oligomer structures as L_2_ or L_10_ appears variable. While mass spectrometry analysis infers a mature L_10_ quaternary structure for MbR^26^ it is uncertain if it forms a comparable toroidal structure to *T. kodakarensis* archaeal Rubisco (PDB: 1GEH), in particular since they only share 36% amino acid homology and MbR contains a novel 11 amino acid insertion in its C-domain^26^. Ongoing efforts are focused on solving the crystal structures for both L_2_ and L_10_ MbR to better understand the structural diversity among archaeal Rubisco as well as help interpret how mutations, such as E138V and K332E, functionally impart changes to catalysis.

## Materials and Methods

### Evolution, expression and purification of MbR in *E. coli*

The *mbii*L gene from pHUE-mbiiL^26^ was cloned into pTrcHisB using *Nco*I/*Hind*III and the resulting pTrcMbR plasmid used as template to randomly mutate the *mbii*L by error-prone PCR (epPCR) as described^20^. The PCR products were cloned into pTrcHisB and the diversity of the *mbii*L mutant library calculated using PEDEL-AA^37^. The library was transformed into the Rubisco Dependent *E. coli* (RDE) strain MM1-*prk* and grown under varying selective conditions according to^20^. The mutant *mbii*L genes from faster growing colonies were cloned into the 6xhistidine-tagged ubiquitin expression plasmid pHUE and each MbR isoform affinity purified by immobilised metal affinity chromatography (IMAC) as described^26^.

### Tobacco plastome transformation and growth

A synthetic gene, *mb*R, coding for *M. burtonii* Rubisco both with and without mutations coding E138 V or K332E substitutions was synthesised by GenScript. The codon use of *mb*R matched the tobacco *rbc*L gene and replaced the native N-terminal coding sequence (MSLIYEDLV) with that for the native tobacco Rubisco large (L-) subunit (MSPQTETKASVGF). The 1,418-bp *mb*R gene fragments were cloned into *Nhe*I/*Sal*I cut pLEV4^32^ to produce the plastome transforming plasmids pLEVmbR, pLEVmbR-K332E and pLEVmbR-E138 V and transformed into ten ^cm^trL leaves by biolistic bombardment^31^. Independent positively transformed lines producing L_10_ MbR were identified by non-denaturing PAGE (native PAGE)^26^ and two independent lines for each MbR genotype were grown to maturity in soil in a growth chamber with the air supplemented with 2.5% (v/v) CO_2_^30^. The flowers of the fertile T_0_ plants were fertilised with wild type pollen and the seed germinated in tissue culture on RMOP media supplemented with 0% to 3% (w/v) sucrose^30^. The germinated T_1_ progeny were carefully transferred to soil and the leaf gas exchange and cell biochemistry of near fully expanded leaves at comparable positions in the upper canopy analysed when the plants were 20–25 cm in height.

### DNA, protein and PAGE analyses

Total leaf genomic DNA was isolated using the DNeasy^®^ Plant Mini Kit and primers LSH and LSE (Fig. 3a) used to PCR amplify and sequence the plastome region transformed in each tobacco genotype as described^32^. The preparation, quantification (against BSA) of soluble leaf protein and analysis by SDS-PAGE, native PAGE and immunoblot analysis was performed as described^38^.

### Rubisco content and catalysis

Rates of Rubisco ^14^CO_2_ fixation were made using soluble protein extracts isolated from bacteria or leaf protein in 50 mM HEPES-NaOH (pH 7.2 or 8.0) containing extraction buffer as described^30^. Protein extract (20 μL) was used to initiate activity in 0.5 mL assays performed in 7 mL septum-capped scintillation vials^38^. Each sample was measured in duplicate under varying concentrations of NaH^14^CO_3_ (0–67 μM) and O_2_ (0, 2, and 5% (v/v)) to calculate the maximal rate of carboxylation (*V*_*C*_) and the Michaelis constants (*K*_*m*_) for CO_2_ (*K*_*C*_) and O_2_ (*K*_*O*_)^38^. The carboxylation turnover rate (*k*_*cat*_^*C*^) was calculated by dividing *V*_*C*_ by the Rubisco active sites content quantified by [^14^C]-2-CABP binding^30^. Rubisco CO_2_/O_2_ specificity (S_C/O_) and the *K*_*m*_ for RuBP were quantified as described^38^ using MbR purified from *E. coli* by immobilised metal affinity chromatography^26^ or from tobacco leaves by ion exchange^30^.

### Growth and photosynthesis analysis

All plants were grown at 25 °C in a growth chamber as described^30^ under 200 ± 50 μmol quanta.m^2^.s^−1^ in air containing 2.5% (v/v) CO_2_. Once approximately 21 cm in height the leaf photosynthesis rates (*A*) in the 5^th^ upper canopy leaf were measured using a LI-6400 XT gas exchange system (LI-COR) at varying atmospheric CO_2_ partial pressures (C_a_; 50–2000 ppm) at a constant leaf temperate of 25 °C and 1000 μmol quanta.m^2^.s^−1^. The “A-C_i_” measurements (C_i_; leaf intercellular CO_2_ levels) were performed at low O_2_ partial pressures (1% (v/v) O_2_ in N_2_) to obtain suitable measures of *A*.

## Additional Information

**How to cite this article**: Wilson, R. H. *et al.* Evolving *Methanococcoides burtonii* archaeal Rubisco for improved photosynthesis and plant growth. *Sci. Rep.* **6**, 22284; doi: 10.1038/srep22284 (2016).

## Supplementary Material

Supplementary Information

## Figures and Tables

**Table 1:** Rubisco catalysis measurements.

Rubisco type	S_C/O_(mol.mol^−1^)	*K*_*C*_(μM)	*k*_*cat*_^*C*^ (s^−1^)	*K*_*O*_(μM)	*k*_*cat*_^*O*^ (s^−1^)	*k*_*cat*_^*C*^/*K*_*C*_(μM.s^−1^)	*k*_*cat*_^*C*^/*K*_*C*_^*21%O2*^ (μM.s^−1^)	*Km*^*RuBP*^ (μM)
MbR	1.3 ± 0.1	56.9 ± 1.8	0.6 ± 0.1	11.2 ± 1.4	0.09	10.5	0.5	4.0 ± 0.5
MbR-E138V	1.5 ± 0.1	66.3 ± 2.1*	1.0 ± 0.1*	35.1 ± 6.1*	0.35	15.1	1.8*	2.0 ± 0.2*
MbR-K332E	1.3 ± 0.1	78.7 ± 2.4*	1.2 ± 0.1*	24.4 ± 4.0*	0.30	15.2	1.4*	4.5 ± 0.8
*R. rubrum*^[1]^	(12)	(149)	(9.0)	(159)	(0.8)	60.4	23.3	(63)
*N. tabacum*^[2]^	(81)	(11)	(3.4)	(259)	(0.8)	309.1	171	(19)

L_10_ MbR catalytic properties at pH 8.0, 25 °C relative to *R. rubrum* L_2_ and tobacco L_8_S_8_ Rubisco. *k*_*cat*_^*O*^, maximal oxygenation rate calculated from S_*C/O*_ = (*k*_*cat*_^*C*^*/K*_*C*_)/(*k*_*cat*_^*O*^*/K*_*O*_). *K*_*C*_^*21%O2*^, *K*_*C*_ under ambient atmospheric O_2_ levels (*O* = 252 μM O_2_ in air saturated H_2_O) calculated as *K*_*C*_(1 + *O*/*K*_*O*_). Values in parenthesis are those measured previously by [1]^20^ and [2]^5^. Significance variation relative to wild type MbR at pH 8.0 (*p < 0.001) determined by T-test.
