# Supplementary material for: Evolving Methanococcoides burtonii archaeal Rubisco for improved photosynthesis and plant growth
Source: Sci Rep. 2016 Mar 1;6:22284. doi: 10.1038/srep22284 (PMC4772096; doi:10.1038/srep22284)
Supplement: Supplementary Information [file srep22284-s1.pdf]

Evolving *Methanococcoides burtonii* archaeal Rubisco for improved photosynthesis and plant growth

Robert H. Wilson, Hernan Alonso, Spencer M. Whitney[*]

Research School of Biology, The Australian National University, Acton, Australian Capital Territory 2601, Australia.

[*]Corresponding author; Research School of Biology, Australian National University, Acton, Australian Capital Territory 2601, Australia; Tel: +61-2-6125-5073; E-mail: spencer.whitney@anu.edu.au

Number of supplemental tables: 1

Number of supplemental figures: 2

Supplemental Table 1. Summary of MbR mutations that improve fitness in the MM1-*prk* RDE screen.

MBR mutant #	*mbii*L mutant library 1	2	3	MbR Amino acid mutations				# silent mutations	PRK selection pressure (% [w/v] arabinose) 1° screen 0.05%	2° screen 0.10%
WT		NA		NA				-	+	-
1, 54	■			K332E				0	++++	++
2	■			V273A	K332I			0	++	-
3	■			H38Q				1	++	-
4	■			T325A				0	+++	+
5	■			T422P				1	++	-
6	■			L372R				2	++	+
7	■			K10I	M76E	I328V		0	++	-
8, 16, 19	■			N251D	T325A			0	++	-
9	■			E179K	T224P	S231L	P304L	0	++	-
10	■			Y448P	E138V			0	+++	+++
10A		*NA		E138V				0	+++	+++
10B				W448P				0	-	-
11	■			E179G				0	++	+
13	■			V369D				0	++	+
14	■			Y471N				0	+++	++
15	■			M423T				0	++	+
17	■			F354S	D364N			0	++	+
18	■			K120I				0	++	+
20	■			D148Y				0	++	+
21, 51	■			K393M				2	++	+
22	■			F295L	P404S			1	++	+
23	■			G327S				0	+++	++
24	■			L377P				1	++	+
25	■			P404A				2	++	+
26	■			L377P				0	++	+
27		■		N300S	D386G			2	++	+
28		■		F99C	G327D			0	+++	+
29		■		L85P	K332E	H353Y	H379N	0	+++	+
30, 31		■		K393R	K406R	P431T		1	+++	+
32		■		A313T	I382T			0	++	+
33		■		A35T				0	++	+
34, 67. 68		■		K393M	P396S			1	++	+
35		■		S344G	T421A	M452V		2	++	+
36, 55	■	■		M423V				0	+++	++
37, 43			■	V20E	A290S	T323A	L407N	0	++	+
38			■	K120E	M128K	E207V		0	++	+
41			■	P239L	K332I			0	+++	+

44			■	T164A	V306A	D385E	0	+++	+
45			■	H379R			0	+++	++
46			■	V398A			1	+++	+
47			■	D376G			0	+++	+
48			■	T325A	E356G		0	++	+
49			■	D141E			0	++	+
50			■	H379P			0	++	+
52			■	D366Y	I382T	F419Y	0	+++	+
53	■			K349E	V369E		0	+++	+
56	■			L464N			2	++	+
57	■			K332E			1	+++	++
58	■			L449H			0	++	+
59	■			V166A			1	++	+
60	■			Y145H	L372P		0	++	+
63		■		T421A			0	+++	++
64		■		K227R	D376Y		0	++	+
65		■		E375K	T421A		0	++	+
66		■		I165T	D418G	V405E	0	++	+
69			■	L162F			0	++	+
70			■	M423I			0	++	+
71			■				3	++	-
72, 75			■	T164P	A326V		1	++	+
73			■	T164P	M423T		0	++	+
74			■	N123R	K332I		1	++	+
76			■	V427G			3	++	+
77			■	E179D	E207G		0	+++	+
78			■	V373A			3	++	+
79			■	T358S			0	++	+
80			■	M248T	I266V	G327S	0	++	+

MM1-*prk* cells (*i.e.* a ΔgapA::Km^r mutated RR1 strain transformed with a pACYC184 plasmid containing the *Synechococcus* PCC7942 *prk*A gene under the control of the BAD promoter) were transformed with three different super-coiled pTrc-*mbii*L mutant libraries generated by epPCR as described[1]. The transformed cells (the primary, 1°, screen) were plated onto M9 selective media that contained 0.4% (v/v) glycerol, 0.05% (w/v) cas amino acids, 0.5 mM IPTG and 0.05% (w/v) L-arabinose containing ampicillin (200 $\mu g.ml^{-1}$) and chloramphenicol (34 $\mu g.ml^{-1}$) as described [1]. Each mutant pTrc- *mbii*L

plasmid was purified, sequenced and transformed back into MM1-*prk* for a secondary (2°) growth screen under higher selective stringency on M9 selective media containing 0.1 % (w/v) L-arabinose. (-) no growth; (+, ++, +++, ++++) relative colony size after 9 days at 23°C in air enriched with 2.5% (v/v) CO_2. MbR mutants supporting the higher RDE fitness (shaded in black) were screened under higher stringency (see Figure 1b in main text). NA, not applicable. **mbii*L mutants coding each separate amino acid mutation from clone #10.

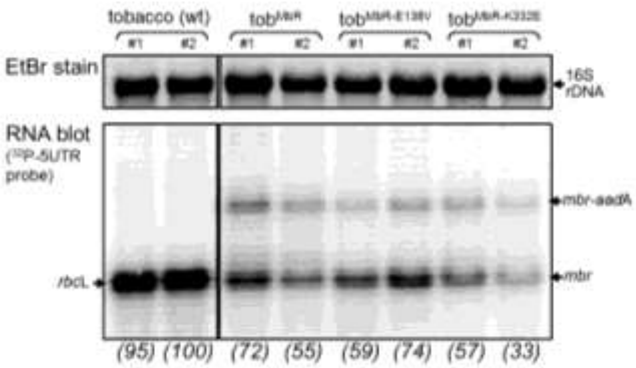

Supplemental Figure 1. Leaf RNA blot analysis.

Total RNA (5µg) extracted from a young upper leaf from replica T_0 genotypes at ~21 cm in height was separated on denaturing formaldehyde gels and the EtBr stained RNA visualised and relative loading confirmed (comparison of 16S rRNA amount in each sample shown). The RNA was blotted onto Hybond-N nitrocellulose membrane (GE healthcare) as described[2] and probed with the ^{32}P-labelled 5UTR probe that comprises the tobacco *rbc*L promoter/5'UTR and first 42 nucleotides of *rbc*L that is conserved in the plastome of each tob^{MbR} genotype (see Fig 3A in main text). The *rbc*L, *mbR* and *mbR-aad*A mRNA transcripts detected by the ^{32}P-5UTR probe are shown with the densitometry measures of total *mbR* mRNA levels relative to the *rbc*L mRNA in WT#2 (shown in italics).

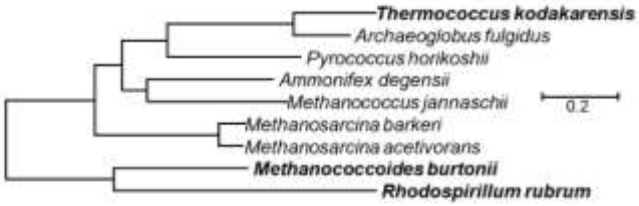

Supplemental Figure 2. MbR sequence phylogeny

Comparative phylogeny of archaeal and *R. rubrum* Rubisco sequences aligned using the Maximum likelihood method by MEGA6. Genbank accessions; *Tk*, WP_011251240; *Af*, WP_010879134; *Ph*, WP_010885029; *Ad*, WP_015739823; *Mj*, B64454; *Mbar*, WP_048176899; *Ma*, WP_011024428; *MbR*, WP_011500311; *Rr*, WP_011390153.

References

1. Mueller-Cajar, O., Morell, M. & Whitney, S.M. Directed evolution of Rubisco in *Escherichia coli* reveals a specificity-determining hydrogen bond in the form II enzyme. *Biochem.* **46**, 14067-74 (2007).
2. Sharwood, R., von Caemmerer, S., Maliga, P. & Whitney, S. The catalytic properties of hybrid Rubisco comprising tobacco small and sunflower large subunits mirror the kinetically equivalent source Rubiscos and can support tobacco growth. *Plant Physiol* **146**, 83-96 (2008).
